# Development of a predictive nomogram for post-liver transplantation complications using clinical parameters and liver stiffness measured by sound touch elastography

**DOI:** 10.1080/07853890.2025.2564928

**Published:** 2025-09-22

**Authors:** Yuan Gao, Bingtian Dong, Ying Wang, Enfa Zhao, Yu Liu, Chaoxue Zhang

**Affiliations:** ^a^ Department of Ultrasound, the First Affiliated Hospital of Anhui Medical University, Hefei, China; ^b^ Department of Nephropathy, the First Affiliated Hospital of Anhui Medical University, Hefei, China; ^c^ Department of Biostatistics and Epidemiology, School of Public Health, Anhui Medical University, Hefei, China

**Keywords:** Nomogram, liver transplantation, complications, liver stiffness measurement, sound Touch elastography

## Abstract

**Background & Aims:**

Monitoring and managing complications after liver transplantation (LT) are crucial for ensuring graft and patient survival. This study aimed to investigate the association between liver stiffness measurement (LSM) and spleen stiffness measurement (SSM) by sound touch elastography (STE) with post-LT complications, and to develop a predictive post-LT complications (PLTC)-nomogram.

**Methods:**

We conducted a retrospective study of patients who received LT between January 2019 and March 2024. After collecting clinical parameters and STE measurements, we constructed a prediction model using univariate and multivariate logistic regression, visualized as a nomogram. Its performance was evaluated with the area under the receiver operating characteristic curve (AUC), precision-recall (PR) curve, calibration curve, and decision curve analysis (DCA). The nomogram’s performance was also compared with LSM, SSM, aspartate aminotransferase-to-platelet ratio index (APRI), and fibrosis-4 index (FIB-4).

**Results:**

A total of 113 recipients were included in the study. Post-LT complications occurred in 41 (36.3%) recipients, including rejection, vascular, biliary, renal, and malignant complications. Multivariate logistic regression analysis identified five factors independently associated with post-LT complications: LSM (odds ratio [OR], 2.64; 95% confidence interval [CI], 1.60–4.35), alkaline phosphatase (OR, 1.02; 95% CI, 1.01–1.04), total bilirubin (OR, 1.08; 95% CI, 1.01–1.15), creatinine (OR, 1.04; 95% CI, 1.02–1.07), and white blood cell count (OR, 0.42; 95% CI, 0.25–0.72). These five factors were used to develop the PLTC-nomogram. The nomogram demonstrated excellent performance with an AUC of 0.968 (95% CI, 0.940–0.996), outperforming LSM (AUC = 0.846), SSM (AUC = 0.676), APRI (AUC = 0.758) and FIB-4 (AUC = 0.800). Area under the PR curve (0.951), calibration curve, and DCA further confirmed that the PLTC-nomogram provided robust diagnostic performance. The PLTC nomogram is available *via* an online platform (https://AYGY-PLTC.shinyapps.io/dynnomapp/).

**Conclusions:**

The PLTC-nomogram incorporating clinical parameters and LSM by STE offers a reliable and noninvasive method for predicting post-LT complications.

## Introduction

Liver transplantation (LT) is the only effective treatment for patients suffering from end-stage liver disease, acute liver failure, and the development of hepatocellular carcinoma (HCC) [1,2]. More than 140,000 LT operations are conducted annually worldwide, with a steadily increasing number of patients on the waiting list. Survival rates for LT recipients have significantly improved over time, with reports indicating one-year survival exceeding 90% and five-year survival around 80% [3]. However, post-operative complications remain the leading cause of mortality in LT recipients [4]. The complications include rejection of the graft, early allograft dysfunction (EAD), vascular complications, biliary tract complications, acute kidney injury (AKI) or progression to chronic kidney disease (CKD), post-transplantation lymphoproliferative disease (PTLD), and recurrence of HCC. Many of these complications are associated with poor survival and diminished quality of life [5–8]. Thus, early identification and management of these complications are crucial for improving both graft and patient survival.

Traditional diagnostic gold standards for post-LT complications include histological biopsy for various post-LT complications (such as graft rejection, PTLD, and recurrence of HCC), endoscopic retrograde cholangiopancreatography (ERCP) for biliary complications, and computed tomography angiography (CTA) for vascular complications. However, histological biopsy and ERCP are both invasive procedures that carry risks of hemorrhage, infection, and other serious complications, and CTA’s contrast agents are often contraindicated in LT patients with hepatic or renal dysfunction. Therefore, there is an urgent need for noninvasive and reliable tools to monitor post-LT complications.

In recent years, ultrasound elastography has gained popularity as a noninvasive method to assess liver condition post-LT. Liver stiffness measurement (LSM) using two-dimensional shear wave elastography (2D-SWE) or vibration-controlled transient elastography (VCTE) is the most commonly used technique. LSM has shown high accuracy in detecting fibrosis in LT recipients and is a valuable marker for predicting post-LT outcomes due to its association with clinical events and mortality [9,10]. Previous studies have demonstrated the utility of LSM in evaluating both allograft damage and biliary complications post-LT [11,12]. Furthermore, spleen stiffness measurement (SSM), another ultrasound elastography biomarker, has been validated as a reliable, noninvasive test for detecting clinically significant portal hypertension [13,14], and also shown predictive value for recurrence of HCC in non-transplanted patients [15]. However, the predictive value of LSM for the risk of overall post-LT complications and the potential application of SSM in the post-LT patients remain to be systematically investigated.

Sound touch elastography (STE) is a new type of 2D-SWE technology. Compared with VCTE and other SWE techniques, STE uses ultra-wideband technology to generate shear waves, which not only provides higher quality elasticity images but also offers more comprehensive quality assessment features, such as motion stability index and reliability maps, helping to improve measurement accuracy [16,17]. STE has demonstrated higher measurement stability and inter-system consistency in multiple studies, which are important aspects contributing to its validity and repeatability [16,18–20]. Despite its promising capabilities, STE has not yet been utilized in the context of LT. Given the complexity of post-LT complications and the need for individualized risk assessment, nomograms that combine clinical and imaging variables into intuitive visual models may facilitate clinical practice in transplant populations. The aim of this study is to explore the association between LSM and SSM by STE and the occurrence of complications after LT, and to develop a predictive post-LT complications (PLTC)-nomogram, which may potentially enhance early risk assessment and guide individualized monitoring strategies for LT recipients.

## Material and methods

### Study design and population

This single-center, retrospective study involved the recipients who underwent LT at the First Affiliated Hospital of Anhui Medical University between January 2019 and March 2024. Participants were included in the study if they met the following criteria: they were aged 18 years or older, had reliable LSM and SSM, and provided detailed ultrasound reports and laboratory tests obtained on the same day as the STE examination. The exclusion criteria were: (1) splenectomy; (2) patients who previously experienced postoperative complications but had recovered; (3) unobtainable or unreliable LSM or SSM; and (4) incomplete clinical data.

Post-LT complications were diagnosed according to standardized criteria: (1) acute and chronic rejection, PTLD, and recurrence of HCC were confirmed by histopathological examination; (2) vascular complications were diagnosed by CTA; (3) biliary complications were confirmed by ERCP; (4) AKI and CKD were diagnosed according to established criteria [21]; and (5) EAD was diagnosed based on standard definition [22]. These complications were confirmed by two transplant hepatologists, with disagreements resolved by a third senior hepatologist.

Based on these diagnostic criteria, patients were classified into complication group and non-complication group. All STE examinations were performed within 48 h before any invasive procedures (such as liver biopsy or ERCP). The complication group included patients with at least one of the above-mentioned conditions confirmed by corresponding gold standards within one week before or after STE examination. The non-complication group consisted of post-LT patients evaluated during the same period without any clinical, radiological, or pathological evidence of complications. The interval from LT to diagnostic assessment represented the time to definitive diagnosis by respective gold standards for patients with complications, and the time to clinical evaluation confirming absence of complications for those without complications.

Our study was approved by the institutional ethics review board of the First Affiliated Hospital of Anhui Medical University (PJ2024-06-54). Written informed consent was obtained from all participants before the commencement of the study, in accordance with the Declaration of Helsinki. The manuscript was reviewed and approved by all authors prior to submission.

### Ultrasound examination and STE measurements

Before the evaluation of the allograft liver, all recipients fasted overnight. A Mindray Resona 7EXP ultrasound system (Mindray, Shenzhen, China) equipped with an SC6-1 convex transducer (Mindray, Shenzhen, China) was used to perform routine postoperative ultrasonographic examination and STE, operated by a sonographer with over 10 years of experience in liver ultrasound and having conducted at least 50 STE examinations. The operator was blinded to the patients’ clinical data and laboratory tests. First, the recipients were assessed through the application of ultrasonography for routine measurements, such as spleen longitudinal diameter and transverse diameter on the maximal sectional image, and hemodynamic parameters of anastomotic vessels. Peak systolic velocity of the hepatic artery (HA-PSV), resistance index of the hepatic artery (HA-RI), maximum velocity of the portal vein (PV-Vmax), and portal vein diameter at the anastomosis (PV-diameter) were recorded.

Next, LSM was performed by STE as follows: patients were placed in a supine position with the right arm completely extended over head. B-mode ultrasound imaging was performed through the right intercostal space, and segment V or VI of the liver was preferentially selected as the target area. In the relatively homogeneous areas of the rectangular elasticity box (4 cm × 3 cm) avoiding vessels and biliary tracts, the region of interest (ROI) with a diameter of 20 mm was placed at least 1–2 cm away from the hepatic surface (Supplemental Figure S1A). During the measurement, patients were asked to hold their breath at the end of expiration for 3–5 s.

The procedure of SSM was analogous to LSM, with the following exceptions: the measurement position was within the left intercostal region, the dimension of the rectangular elastic box was (1.5 cm × 1.5 cm), and the ROI diameter was reduced to 10 mm (Supplemental Figure S1B).

For both LSM and SSM, five successful acquisitions were performed for each patient, and median values were calculated and recorded according to the quality criteria provided by the manufacturer. A valid measurement was required to meet the following conditions simultaneously: (1) The signal contained at least two-thirds within the rectangular elasticity box; (2) The image revealed a reliability index higher than 90%; and (3) The presented motion stability index attained a level of no less than four stars. Reliable measurements were characterized as presenting an interquartile range (IQR)-to-median (IQR/M) less than 30% [23].

### Serum tests

Laboratory tests were performed on the same day as the STE examinations. Blood routine, liver and kidney function tests, and coagulation function analyses were conducted, and the corresponding biochemical parameters were recorded. Based on these biochemical indicators, noninvasive fibrosis scores, including aspartate aminotransferase-to-platelet ratio index (APRI) and fibrosis-4 index (FIB-4) [24,25], were calculated and recorded.

### Statistical analysis

For continuous variables, when the data were normally distributed, they were presented as mean ± standard deviation; otherwise, they were shown as median and IQR. For categorical variables, data were displayed as numbers and proportions. To compare the characteristics of patients with and without post-LT complications, Fisher’s exact test was applied for categorical data, and the Mann-Whitney *U* test was used for continuous variables. To identify the predictors associated with complications after LT, univariate and multivariate logistic regression analyses were employed, with LSM and SSM analyzed as continuous variables. No adjustments for multiple comparisons were applied to the univariate analysis, as this was used for exploratory variable selection prior to multivariate analysis. The multivariate analysis included all variables with a *P* value of less than 0.1 in the univariate logistic regression analysis. Finally, the results were used to construct a nomogram, which was evaluated in terms of discrimination, calibration, and clinical usefulness. Given the sample size constraints, we implemented ten-fold cross-validation for internal validation instead of performing a separate training/validation data split to assess model performance and reduce overfitting risk. Discrimination was assessed using the area under the receiver operating characteristic curve (AUC) and area under the precision-recall curve (PR-AUC). Model calibration was evaluated using both the calibration curve and the Hosmer-Lemeshow goodness-of-fit test. Decision curve analysis (DCA) was performed to determine clinical utility. The predictive performance of the nomogram was compared with LSM, SSM, FIB-4, and APRI using the Delong test. Additionally, to further assess the model’s performance across different clinical scenarios, we conducted subgroup analyses based on HCC history, evaluating the nomogram’s predictive ability, calibration, and clinical utility separately in patients with and without a history of HCC using the same methodological approaches described above. All statistical analyses were performed using SPSS version 26.0 (SPSS, Chicago, IL, USA) and R software version 4.3.1 (R Foundation for Statistical Computing, Vienna, Austria). *p* < 0.05 was considered statistically significant.

## Results

### Patient characteristics

**Figure 1.**
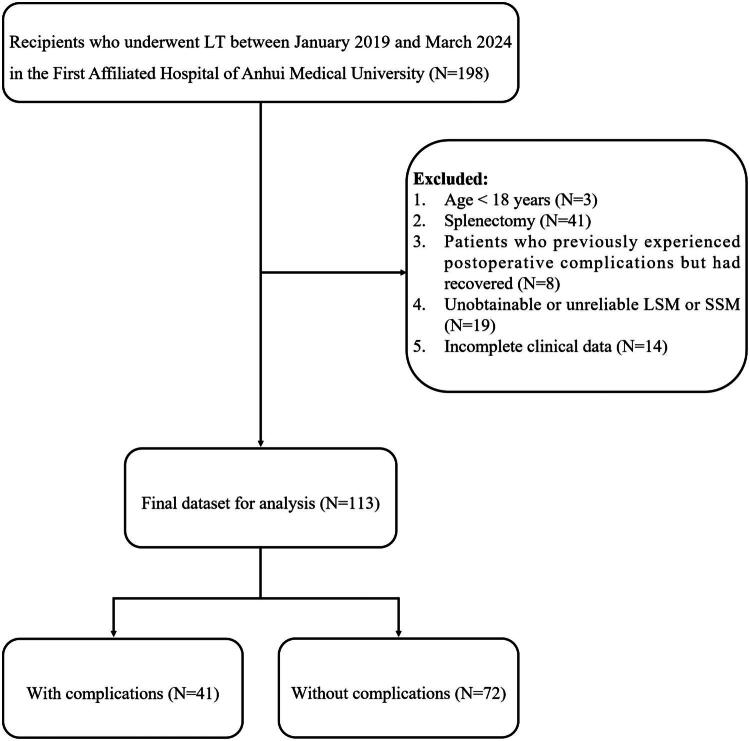
Flowchart of the study. LT: liver transplantation; STE: sound touch elastography; LSM: liver stiffness measurement; SSM: spleen stiffness measurement.

As shown in the study flowchart (Figure 1), among the 198 recipients initially screened, a total of 113 subjects who met the inclusion criteria were enrolled. Post-LT complications occurred in 41 out of the 113 (36.3%) recipients. These complications included acute or chronic allograft rejection (*N* = 8), hepatic artery stenosis (*N* = 6), hepatic artery thrombosis (*N* = 2), portal vein thrombosis (*N* = 4), stenosis or stones of biliary tracts (*N* = 13), bile leak (*N* = 1), AKI and CKD (*N* = 11), PTLD (*N* = 1), EAD (*N* = 1), and recurrence of HCC (*N* = 2). Eight patients experienced two different types of complications simultaneously. Among the 41 patients, three patients died: one due to vein thrombosis combined with heart failure, another due to EAD, and the third as a result of the coexistence of hepatic artery thrombosis and bile leakage.

The baseline demographic, clinical, biochemical, and ultrasound characteristics of the included patients are summarized in Table 1. The median interval from LT to diagnostic assessment was 56 days (IQR, 12–545 days). The mean age was 50.82 ± 10.81 years with a male predominance of 76.1%. The mean BMI was 22.97 ± 3.12 kg/m^2^. The most common indication for LT was viral hepatitis (65.5%), and 36.3% of patients had a history of HCC. Regarding the APRI and FIB-4 index, the median values were 0.59 (IQR, 0.32–1.56) and 2.44 (IQR, 1.34–4.78), respectively.

**Table 1.** Baseline demographic, clinical, biochemical and ultrasound characteristics of the enrolled population (*N* = 113).

Variables	All (*N* = 113)	Patients with complications (*N* = 41)	Patients without complications (*N* = 72)	*p* Value
Age (years)	50.82 ± 10.81	52.24 ± 9.98	50.01 ± 11.24	0.294
Male, n (%)	86 (76.1)	31 (75.6)	55 (76.4)	0.926
BMI (kg/m^2^)	22.97 ± 3.12	22.60 ± 2.98	23.18 ± 3.19	0.341
Etiology, n (%)				0.849
HBV/HCV	74 (65.5)	25 (61.0)	49 (68.1)	
PBC/PSC/AIH	13 (11.5)	5 (12.2)	8 (11.1)	
Alcoholic	13 (11.5)	5 (12.2)	8 (11.1)	
*Others	13 (11.5)	6 (14.6)	7 (9.7)	
History of HCC, n (%)	41 (36.3)	16 (39.0)	25 (34.7)	0.647
Hypertension, n (%)	5 (4.4)	3 (7.3)	2 (2.8)	0.351
Diabetes, n (%)	16 (14.2)	6 (14.6)	10 (13.9)	0.913
LSM (kPa)	10.93 ± 2.87	13.14 ± 2.50	9.67 ± 2.25	< 0.001
SSM (kPa)	17.91 (15.38, 20.32)	19.51 (16.56, 23.26)	16.90 (13.78, 19.18)	0.002
Spleen longitudinal diameter (cm)	13.8 (11.8, 16.2)	14.9 (12.6, 16.9)	13.1 (11.6, 16.0)	0.009
Spleen transverse diameter (cm)	4.5 (3.9, 5.4)	4.7 (4.1, 5.6)	4.4 (3.7, 5.3)	0.025
HA-PSV (cm/s)	65 (54, 80)	60 (42, 74)	68 (56, 85)	0.007
HA-RI	0.68 (0.62, 0.74)	0.69 (0.61, 0.76)	0.68 (0.62, 0.72)	0.672
PV-V_max_ (cm/s)	42 (30, 58)	36 (28, 49)	44 (32, 66)	0.046
PV-diameter (cm)	0.75 (0.70, 0.86)	0.77 (0.72, 0.89)	0.75 (0.69, 0.86)	0.481
ALT (IU/L)	31.00 (16.30, 72.00)	35.00 (15.00, 95.00)	29.95 (16.78, 62.95)	0.418
AST (IU/L)	26.00 (17.00, 44.50)	42.90 (21.00, 99.30)	23.50 (16.93, 33.83)	< 0.001
ALP (IU/L)	114.00 (87.00, 193.00)	159.10 (98.90, 214.00)	100.50 (84.72, 143.03)	< 0.001
GGT (IU/L)	66.00 (26.7, 131.00)	100.00 (29.00, 197.60)	57.00 (23.75, 114.93)	0.034
ALB (g/L)	41.47 ± 4.36	40.81 ± 4.11	41.85 ± 4.48	0.225
TBIL (µmol/L)	21.60 (15.30, 35.80)	27.90 (18.10, 55.70)	19.71 (13.92, 27.02)	< 0.001
CRE (µmol/L)	74.00 (60.80, 104.00)	104.00 (69.20, 181.00)	68.85 (57.48, 90.50)	0.001
PLT (10^9^/L)	106.00 (77.00, 166.00)	87.00 (62.00, 109.00)	120.00 (88.00, 181.75)	< 0.001
WBC (10^9^/L)	5.35 (3.78, 7.47)	4.29(3.29, 5.97)	5.89 (4.13, 7.90)	0.009
HB (g/L)	111.00 (95.00, 137.00)	112.00 (97.00, 136.00)	108.00 (94.00, 138.50)	0.974
INR	1.02 (0.98, 1.05)	1.02 (0.98, 1.06)	1.02 (0.98, 1.04)	0.407
APRI	0.59 (0.32, 1.56)	1.48 (0.67, 2.93)	0.46 (0.29, 0.85)	< 0.001
FIB-4	2.44 (1.34, 4.78)	4.84 (2.70, 8.87)	1.91 (1.05, 2.91)	< 0.001

*Note*. Data are presented as n (%), mean ± standard deviation, or median (IQR).

* Including acute drug-induced liver failure, hepatic veno-occlusive disease, schistosomiasis, glycogen storage disease, and polycystic liver.

Abbreviations: BMI: body mass index; HBV: hepatitis B virus; HCV: hepatitis C virus; PBC: primary biliary cholangitis; PSC: primary sclerosing cholangitis; AIH: autoimmune hepatitis; HCC: hepatocellular carcinoma; HA-PSV: peak systolic velocity of hepatic artery; RI: resistance index; PV-Vmax: maximum velocity of the portal vein; ALT: alanine aminotransferase; AST: aspartate aminotransferase; ALP: alkaline phosphatase; GGT: γ-glutamyltransferase; ALB: albumin; TBIL: total bilirubin; CRE: creatinine; PLT: platelet count; WBC: white blood cell count; HB: hemoglobin; INR: international normalized ratio; APRI: aspartate aminotransferase-to-platelet ratio index; FIB-4: fibrosis-4; LSM: liver stiffness measurement; SSM: spleen stiffness measurement.

### Comparison of clinical and ultrasonographic parameters of patients with and without post-LT complications

The mean LSM value was higher in participants with complications compared to those without (13.14 kPa vs. 9.67 kPa; *p <* 0.001) (Supplemental Figure S2A). Similarly, the median SSM value was higher in participants with complications than in those without (19.51 kPa vs. 16.90 kPa; *p =* 0.002) (Supplemental Figure S2B). The median spleen longitudinal diameter (14.9 cm vs. 13.1 cm; *p =* 0.009) and transverse diameter (4.7 cm vs. 4.4 cm; *p =* 0.025) were higher in patients with complications. The median HA-PSV (60 cm/s vs. 68 cm/s; *p =* 0.007) and PV-Vmax (36 cm/s vs. 44 cm/s; *p =* 0.046) were lower in patients with complications. The APRI (1.48 vs. 0.46; *p* < 0.001) and FIB-4 (4.84 vs. 1.91; *p* < 0.001) were higher in patients with complications.

Patients with post-LT complications had higher median levels of aspartate aminotransferase (AST, 42.90 IU/L vs. 23.50 IU/L, *p* < 0.001), alkaline phosphatase (ALP, 159.10 IU/L vs. 100.50 IU/L, *p* < 0.001), γ-glutamyltransferase (GGT, 100.00 IU/L vs. 57.00 IU/L, *p* = 0.034), total bilirubin (TBIL, 27.90 µmol/L vs. 19.71 µmol/L, *p* < 0.001), creatinine (CRE, 104.00 µmol/L vs. 68.85 µmol/L, *p* = 0.001), and lower platelet count (PLT; 87.00 × 10^9^/L vs. 120.00 × 10^9^/L; *p* < 0.001) and white blood cell count (WBC; 4.29 × 10^9^/L vs. 5.89 × 10^9^/L; *p* = 0.009).

### Factors associated with post-LT complications

In univariate analysis, spleen longitudinal diameter, AST, ALP, TBIL, PLT, WBC, LSM, and SSM were associated with post-LT complications (*p* < 0.1). In multivariate logistic analysis, LSM (odds ratio [OR], 2.64; 95% confidence interval [CI], 1.60–4.35), ALP (OR, 1.02; 95% CI, 1.01–1.04), TBIL (OR, 1.08; 95% CI, 1.01–1.15), CRE (OR, 1.04; 95% CI, 1.02–1.07) and WBC (OR, 0.42; 95% CI, 0.25–0.72) were independently associated with complications after LT (Table 2).

**Table 2.** Univariate and multivariate analyses of variables associated with post-transplantation complications (*N* = 113).

Variables	Univariable analysis			Multivariable analysis		
	OR	95%CI	*p* Value	OR	95%CI	*p* Value
Age	1.02	0.98–1.06	0.291			
Male	0.96	0.39–2.35	0.926			
BMI	0.94	0.83–1.07	0.339			
Etiology						
HBV	1.00	Reference				
PBC/PSC/AIH	1.22	0.36–4.14	0.744			
Alcohol	1.22	0.36–4.14	0.744			
*Others	1.68	0.51–5.53	0.394			
History of HCC	1.20	0.54–2.66	0.648			
Hypertension	2.76	0.44–17.26	0.277			
Diabetes	1.06	0.36–3.17	0.913			
LSM	1.90	1.47–2.44	<.001	2.64	1.60–4.35	<.001
SSM	1.15	1.05–1.26	0.004			
Spleen longitudinal diameter	1.02	1.01–1.03	0.026			
Spleen transverse diameter	1.04	1.00–1.08	0.053			
HA-PSV	0.98	0.97–1.00	0.079			
HA-RI	0.37	0.01–12.36	0.576			
PV-Vmax	1.00	0.99–1.01	0.938			
PV-diameter	1.13	0.87–1.47	0.357			
ALT	1.00	1.00–1.01	0.197			
AST	1.01	1.01–1.02	0.011			
ALP	1.01	1.01–1.01	0.003	1.02	1.01–1.04	0.032
GGT	1.00	1.00–1.01	0.050			
ALB	0.95	0.86–1.03	0.224			
TBIL	1.02	1.01–1.04	0.008	1.08	1.01–1.15	0.041
CRE	1.02	1.01–1.03	<.001	1.04	1.02–1.07	0.001
PLT	0.99	0.98–0.99	0.001			
HB	1.00	0.98–1.01	0.775			
WBC	0.83	0.72–0.96	0.015	0.42	0.25–0.72	0.001
INR	4.64	0.34–62.67	0.248			

* Including acute drug-induced liver failure, hepatic veno-occlusive disease, schistosomiasis, glycogen storage disease, and polycystic liver.

Abbreviations: BMI: body mass index; HBV: hepatitis B virus; HCV: hepatitis C virus; PBC: primary biliary cholangitis; PSC: primary sclerosing cholangitis; AIH: autoimmune hepatitis; HCC: hepatocellular carcinoma; HA-PSV: peak systolic velocity of hepatic artery; RI: resistance index; PV-Vmax: maximum velocity of the portal vein; ALT: alanine aminotransferase; AST: aspartate aminotransferase; ALP: alkaline phosphatase; GGT: γ-glutamyltransferase; ALB: albumin; TBIL: total bilirubin; CRE: creatinine; PLT: platelet count; WBC: white blood cell count; HB: hemoglobin; INR: international normalized ratio; APRI: aspartate aminotransferase-to-platelet ratio index; FIB-4: fibrosis-4; LSM: liver stiffness measurement; SSM: spleen stiffness measurement; OR: odd ratio; CI: confidence interval.

### Development of the PLTC-nomogram for predicting post-LT complications

**Figure 2.**
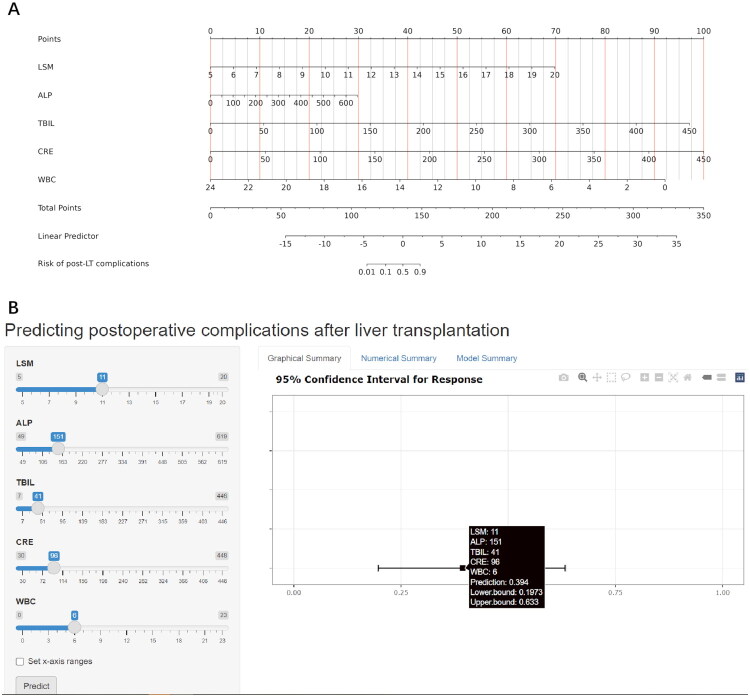
The PLTC-nomogram and its web-based application for predicting post-transplantation complications. (A) The PLTC-nomogram for risk assessment of post-transplantation complications. (B) Interface of the web-based nomogram calculator (accessible at https://AYGY-PLTC.shinyapps.io/dynnomapp/). A case example is demonstrated: for a patient with LSM of 11 kPa, ALP of 151 IU/L, TBIL of 41 µmol/L, CRE of 96 µmol/L, and WBC of 6 × 10^9^/L, the predicted probability of developing post-transplantation complications is 39.4%. PLTC: post-liver transplantation complication; LSM: liver stiffness measurement; ALP: alkaline phosphatase; TBIL: total bilirubin; CRE: creatinine; WBC: white blood cell count.

**Figure 3.**
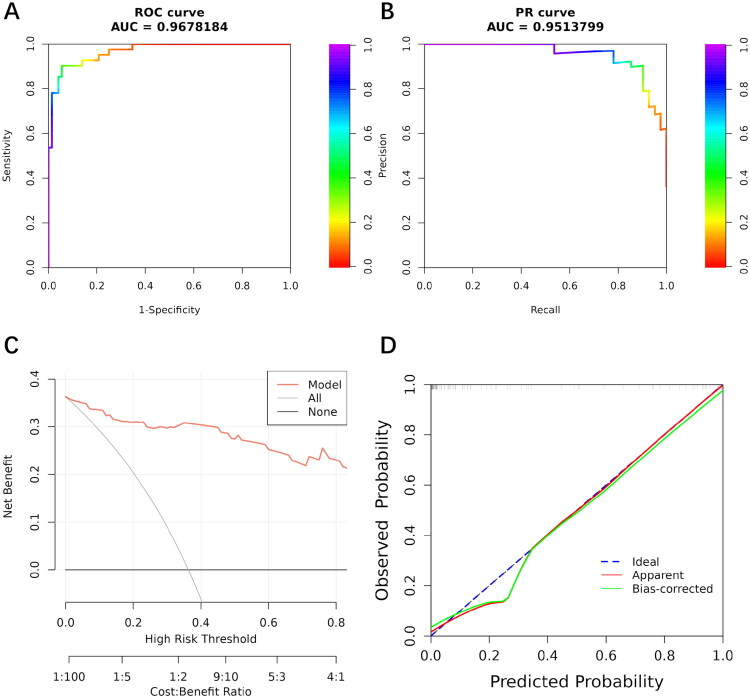
Performance of the PLTC-nomogram model. (A) Receiver operating characteristic (ROC) curve analysis of the nomogram. (B) Precision-recall (PR) curve of the nomogram. (C) Decision curve analysis (DCA). (D) Calibration curves. PLTC: post-liver transplantation complication.

The above five factors were integrated into a logistic regression model and presented as the PLTC-nomogram (Figure 2(A)). To facilitate clinical application, we developed an online calculator (https://AYGY-PLTC.shinyapps.io/dynnomapp/) that provides instant risk assessment, as demonstrated in Figure 2(B). The PLTC-nomogram exhibited outstanding discriminative ability with an AUC of 0.968 (95% CI, 0.940–0.996) (Figure 3(A)). The precision-recall (PR) curve analysis further validated the model’s excellent performance, achieving a PR-AUC of 0.951 and maintaining high precision across varying recall thresholds (Figure 3(B)). DCA demonstrated the model’s clinical utility by showing consistent positive net benefits across threshold probabilities from 0 to 0.8, superior to both “treat all” and “treat none” strategies (Figure 3(C)). The calibration curve indicated optimal agreement between predicted and observed probabilities (Figure 3(D)), which was further supported by the Hosmer-Lemeshow test (*p* = 0.950).

### Performance of the PLTC-nomogram, LSM, SSM, FIB-4 and APRI for predicting post-LT complications

**Figure 4.**
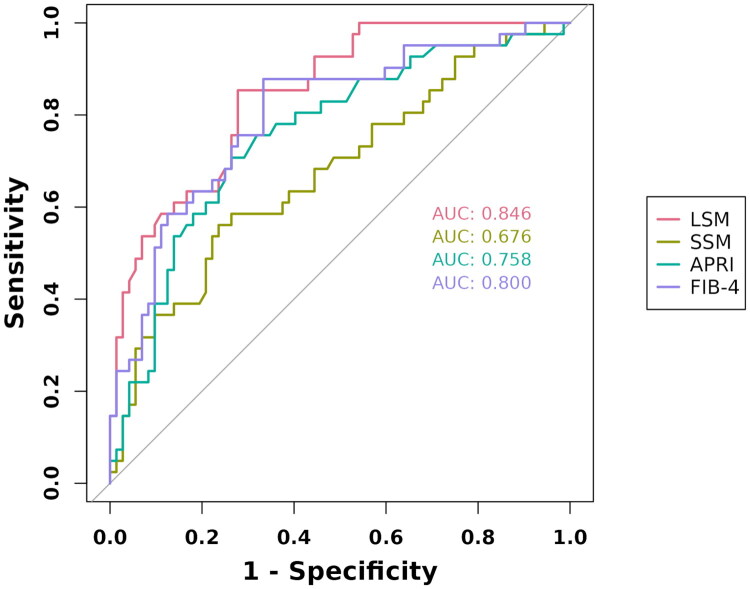
ROC curves comparing the diagnostic performance of LSM, SSM, APRI, and FIB-4 for predicting post-liver transplantation complications. LSM, liver stiffness measurement; SSM, spleen stiffness measurement; APRI, aspartate aminotransferase-to-platelet ratio index; FIB-4, fibrosis-4.

At the optimal cutoff value of 0.40, the PLTC-nomogram achieved excellent predictive performance with high sensitivity (94.4%) and specificity (90.2%), along with optimal positive and negative predictive values (94.4% and 90.2%, respectively). This performance substantially outperformed other noninvasive methods: LSM (AUC = 0.846, 95% CI: 0.776–0.917), FIB-4 (AUC = 0.800, 95% CI: 0.713–0.886), APRI (AUC = 0.758, 95% CI: 0.664–0.852), and SSM (AUC = 0.676, 95% CI: 0.572–0.781) (Table 3, Figure 4).

**Table 3.** Diagnostic performances of PLTC-nomogram, LSM, SSM, APRI, and FIB-4 for predicting post-transplantation complications.

	Cutoff Value	AUROC (95% CI)	Sensitivity (%)	Specificity (%)	PPV (%)	NPV (%)
PLTC-nomogram	0.40	0.968 (0.940–0.996)	94.4	90.2	94.4	90.2
LSM	10.93 kPa	0.846 (0.776–0.917)	85.4	72.2	63.6	89.7
SSM	19.21 kPa	0.676 (0.572–0.781)	56.1	76.4	57.5	75.4
APRI	0.71	0.758 (0.664–0.852)	70.7	73.6	60.4	81.5
FIB-4	2.19	0.800 (0.713–0.886)	87.8	66.7	60.0	90.6

Abbreviations: PLTC: post-liver transplantation complications; LSM: liver stiffness measurement; SSM: spleen stiffness measurement; APRI: aspartate aminotransferase-to-platelet ratio index; FIB-4: fibrosis-4; AUROC: area under the receiver operating characteristic curve; CI: confidence interval; PPV: positive predictive value; NPV: negative predictive value.

### Subgroup analysis based on history of HCC

To further evaluate the predictive performance of the PLTC-nomogram in different clinical settings, we conducted a subgroup analysis in patients with HCC history (*N* = 41, 36.3%) and those without (*N* = 72, 63.7%). The PLTC-nomogram maintained excellent predictive ability in both subgroups. For patients with a history of HCC (Supplemental Figure S3), the model demonstrated an AUC of 0.985 (95% CI, 0.957–1.000). The PR curve, calibration curve, and DCA also confirmed robust performance in this subgroup. Similarly, in patients without HCC history (Supplemental Figure S4), the model achieved an AUC of 0.965 (95% CI, 0.929–1.000), with supplementary analytical curves further validating its reliability and clinical utility in this subgroup.

## Discussion

Our study demonstrated that LSM by STE was significantly associated with post-LT complications and served as an independent and incremental predictor, whereas SSM did not demonstrate such predictive value. Subsequently, we developed a novel PLTC-nomogram incorporating LSM and four clinical parameters for predicting post-LT complications. The PLTC-nomogram exhibited excellent discriminative performance (AUC = 0.968, PR-AUC = 0.951), superior to LSM, SSM, APRI, and FIB-4 (AUCs = 0.846, 0.676, 0.758, and 0.800, respectively), demonstrating robust clinical utility.

The significant predictive value of LSM can be explained by its unique ability to directly reflect pathophysiological changes in liver grafts. Post-LT complications, whether vascular, biliary, or immunological, often lead to parenchymal alterations that manifest as changes in liver stiffness before biochemical abnormalities become apparent [11,12]. This mechanical property change occurs through various mechanisms, including inflammatory cell infiltration, edema, congestion, early fibrotic changes [26]. Traditional serum-based indices such as APRI and FIB-4 primarily reflect hepatic inflammation and fibrosis indirectly through surrogate markers. Similarly, SSM functions as an indirect indicator by measuring portal pressure, whereby its variations reflect both intrahepatic and extrahepatic factors that influence portal pressure [13]. In contrast, LSM provides real-time, direct measurement of tissue elasticity. Consequently, this fundamental difference in measurement approach may explain why LSM demonstrated superior predictive performance compared to APRI, FIB-4 and SSM in our study. Previous studies have demonstrated the utility of LSM in evaluating various post-LT complications, including biliary complications, acute rejection and EAD [12,27,28]. Unlike these focused investigations, our study represents the first systematic evaluation integrating LSM with conventional biochemical parameters into a comprehensive predictive model (AUC = 0.968), which substantially outperforms both LSM (AUC = 0.846) and SSM (AUC = 0.676).

Beyond the role of LSM, ALP was demonstrated to be independently associated with post-LT complications. The predictive role of ALP is not limited to indicating cholestasis but also serves as an important biomarker for graft dysfunction and biliary complications following LT [29,30]. In our cohort, biliary complications represented 31.7% (13/41) of all documented post-LT complications, highlighting the clinical relevance of this marker. The elevation of ALP after LT may reflect underlying inflammatory processes affecting the biliary epithelium or early signs of rejection. By identifying ALP as an independent prognostic factor for survival outcomes after LT, Wannhoff et al. [29] highlighted its potential as a noninvasive marker for predicting survival and graft failure risk in LT recipients.

Similar to ALP, TBIL is a multifaceted marker that reflects not only cholestasis but also hepatocellular function and biliary excretion capacity, making it valuable in comprehensive liver assessment. Persistent hyperbilirubinemia beyond postoperative day 7 (>10 mg/dL) has been established as one of the three diagnostic criteria for EAD, associated with significantly increased risk of graft failure and mortality [31]. Beyond biliary complications and recurrence of biliary diseases, The elevation of bilirubin can manifest in various post-LT pathological processes [32,33], its limited specificity necessitates comprehensive clinical evaluation for accurate diagnosis.

Moreover, the results also showed that increased serum CRE was independently associated with an increased risk of postoperative complications. Elevated serum CRE level is a marker of impaired kidney function. Kidney function deterioration after LT is mainly due to the nephrotoxicity of immunosuppressive medications, pretransplant factors, and the development of metabolic syndrome. Pre-LT serum CRE is also regarded as an independent risk factor for 90-day mortality after LT [34], and variation of serum CRE is significantly associated with pre- and post-LT outcomes and mortality [35].

Interestingly, we found that decreased WBC count was associated with increased complication risk, the reduced WBC levels may be attributed to hypersplenism. Our findings demonstrated that patients in the complication group exhibited significantly larger longitudinal and transverse diameters of spleen compared to those in the non-complication group. Furthermore, the complication group showed markedly lower levels of WBC, PLT, and HB than the non-complication group. These observations suggest that the development of complications may be associated with increased splenic dimensions, although this hypothesis warrants further investigation. An alternative explanation could be that patients in the complication group may experience excessive immunosuppression, potentially compromising the body’s immune surveillance against viral infections and tumor cells, as evidenced in cases of PTLD and HCC recurrence [36]. The combination of these parameters in our model provides a more comprehensive evaluation than single markers reported in previous studies.

To further assess our PLTC-nomogram’s generalizability, we performed subgroup analysis between HCC and non-HCC recipients. The model maintained exceptional predictive performance in both HCC (AUC = 0.985) and non-HCC recipients (AUC = 0.965). Despite varying disease mechanisms and pre-LT status, the remarkable consistency across subgroups indicates our parameters likely reflect fundamental biological processes potentially common among diverse LT recipient populations. These findings provide initial evidence for the nomogram’s potential broader applicability across different clinical settings.

This investigation has several notable strengths. First, it represents the first use of STE technology to predict post-LT complications. STE offers advantages including higher quality elasticity images through ultra-wideband technology and comprehensive quality assessment features, which are particularly valuable considering the challenges inherent to post-LT measurements. Second, we simultaneously evaluated liver and spleen stiffness, not only investigating the association of LSM and SSM with post-LT complications but also comparing their predictive values, which has not been previously reported. Third, we integrated STE measurements and clinical parameters to establish a prediction model, which demonstrated superior predictive performance compared to using individual methods alone.

However, our study also presented several limitations that deserve mention. First, as a single-center retrospective study, inherent selection bias might exist. To minimize potential selection bias, predefined inclusion and exclusion criteria were implemented before patient enrollment. All procedures were performed by the same sonographer using specific ultrasound equipment from a single vendor, to minimize other factors related to ultrasound measurements. Second, our preliminary exploration with a relatively small sample (*N* = 113) lacks external validation, potentially risking overfitting and overestimated performance metrics. Despite the model’s promising predictive performance, future prospective, multicenter studies across diverse liver transplant centers are needed to evaluate the nomogram’s generalizability and clinical utility in varied patient populations. Comprehensive demographic representation will be essential to confirm the model’s robustness for widespread clinical application. Third, the limited sample size prevented detailed subgroup analyses for specific complications or recipient characteristics. Although our preliminary subgroup analysis showed comparable diagnostic performance between HCC and non-HCC recipients, larger studies are needed to validate our nomogram’s predictive value across different recipient populations, including those with varied underlying diseases and specific types of post-LT complications. Fourth, as liver transplant recipients are still under follow-up, survival analysis was not included in the present study. Future investigations are warranted to validate the utility of this nomogram in predicting post-LT mortality.

## Conclusion

In conclusion, we developed a novel prediction model that effectively integrates LSM by STE with clinical parameters for prediction of post-LT complications, exhibiting excellent discriminative ability and robust clinical utility. By providing a reliable and noninvasive approach, our model holds promise for guiding individualized postoperative management strategies and potentially improving patient outcomes.

## Supplementary Material

Supplementary materials.docx

## Data Availability

The dataset is available from the corresponding author, Prof. Chaoxue Zhang, with permission from the First Affiliated Hospital of Anhui Medical University and upon justifiable request.
