# Supplementary material for: Development of a predictive nomogram for post-liver transplantation complications using clinical parameters and liver stiffness measured by sound touch elastography
Source: Ann Med. 2025 Sep 22;57(1):2564928. doi: 10.1080/07853890.2025.2564928 (PMC12459177; doi:10.1080/07853890.2025.2564928)

**Figure S1**. Liver and spleen stiffness measurements by sound touch elastography. (**A**) The dimension of the rectangular elastic box was(4 cm×3 cm), and the ROI was 20 mm. (**B**) The dimension of the rectangular elastic box was(1.5 cm×1.5 cm), and the ROI was 10 mm. The reliability indexes were 100%, and the motion stability indexes were 5 stars for both.


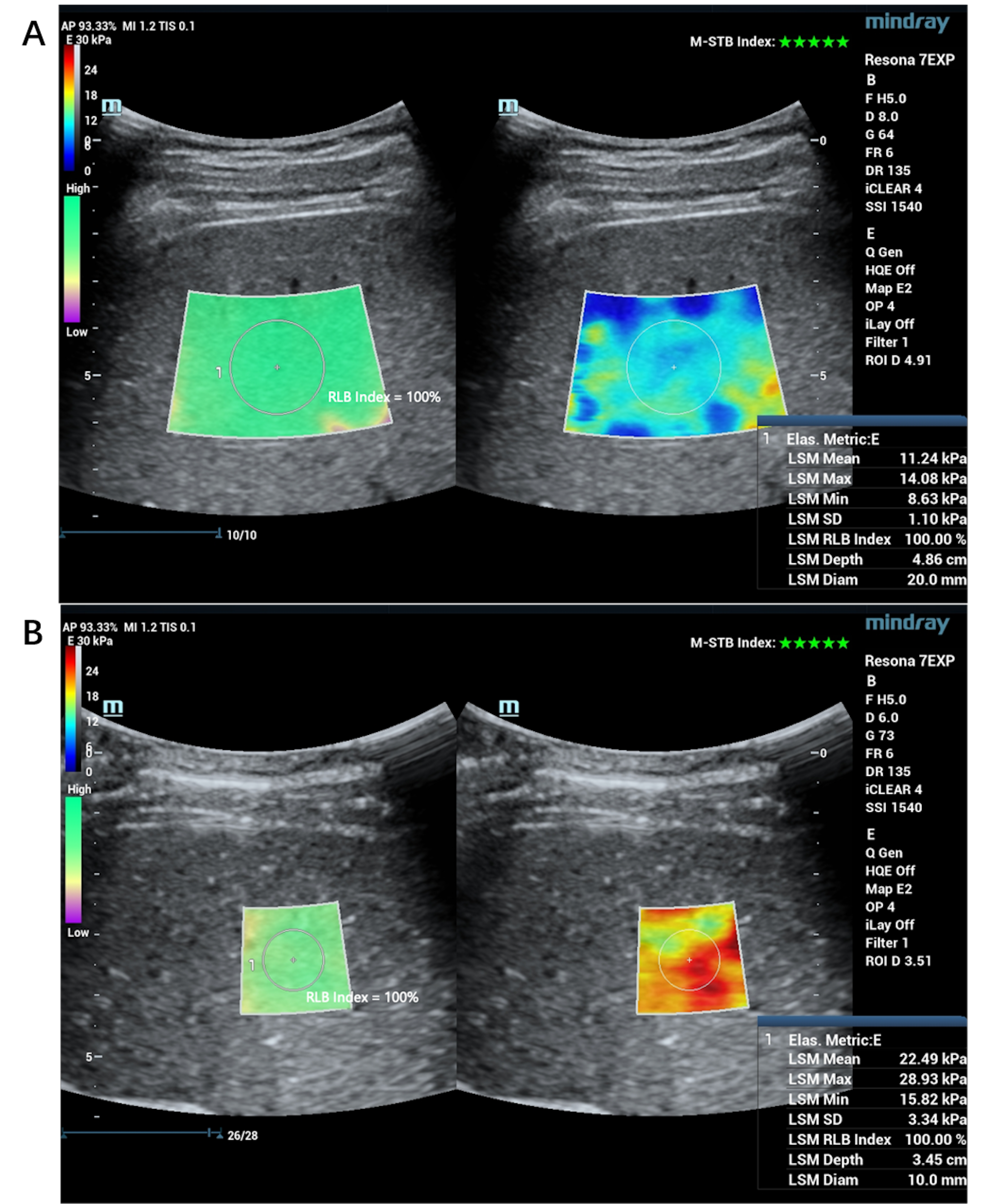


**Figure S2**. Comparison of LSM and SSM between patients with and without post-transplantation complications. (**A**) The box represents the standard deviation and the middle line represents the mean value of LSM. The LSM value was significantly higher in patients with complications (*P <* 0.001). (**B**) The box represents the interquartile range (25th-75th percentile) and the line in the box represents the median value of SSM. The SSM value was significantly higher in patients with complications (*P =* 0.002).


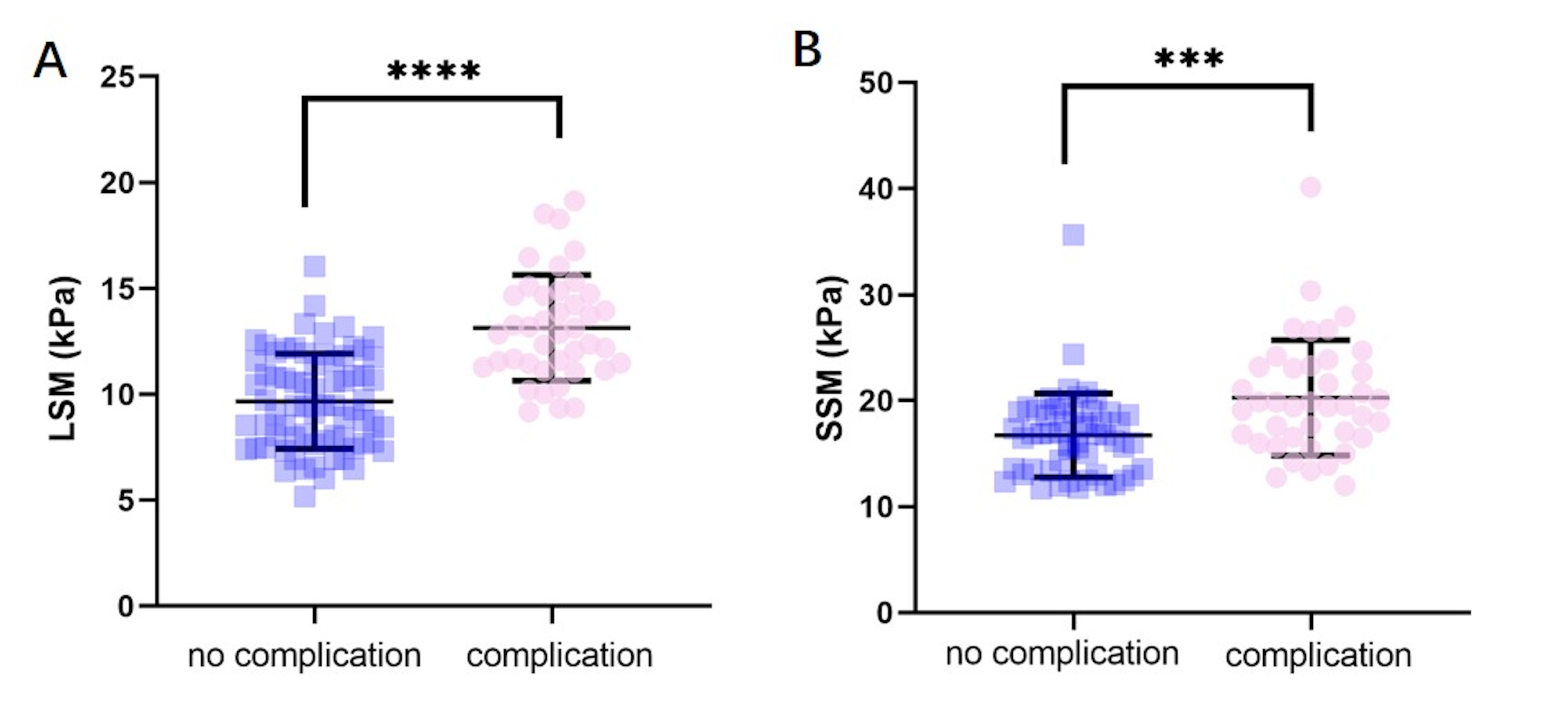


**Figure S3.** Performance of the PLTC-nomogram in patients with HCC history (N=41). (**A**) Receiver operating characteristic (ROC) curve analysis of the nomogram. (**B**) Precision-recall (PR) curve of the nomogram; (**C**) Decision curve analysis (DCA). (**D**) Calibration curves. PLTC, post-liver transplantation complication.


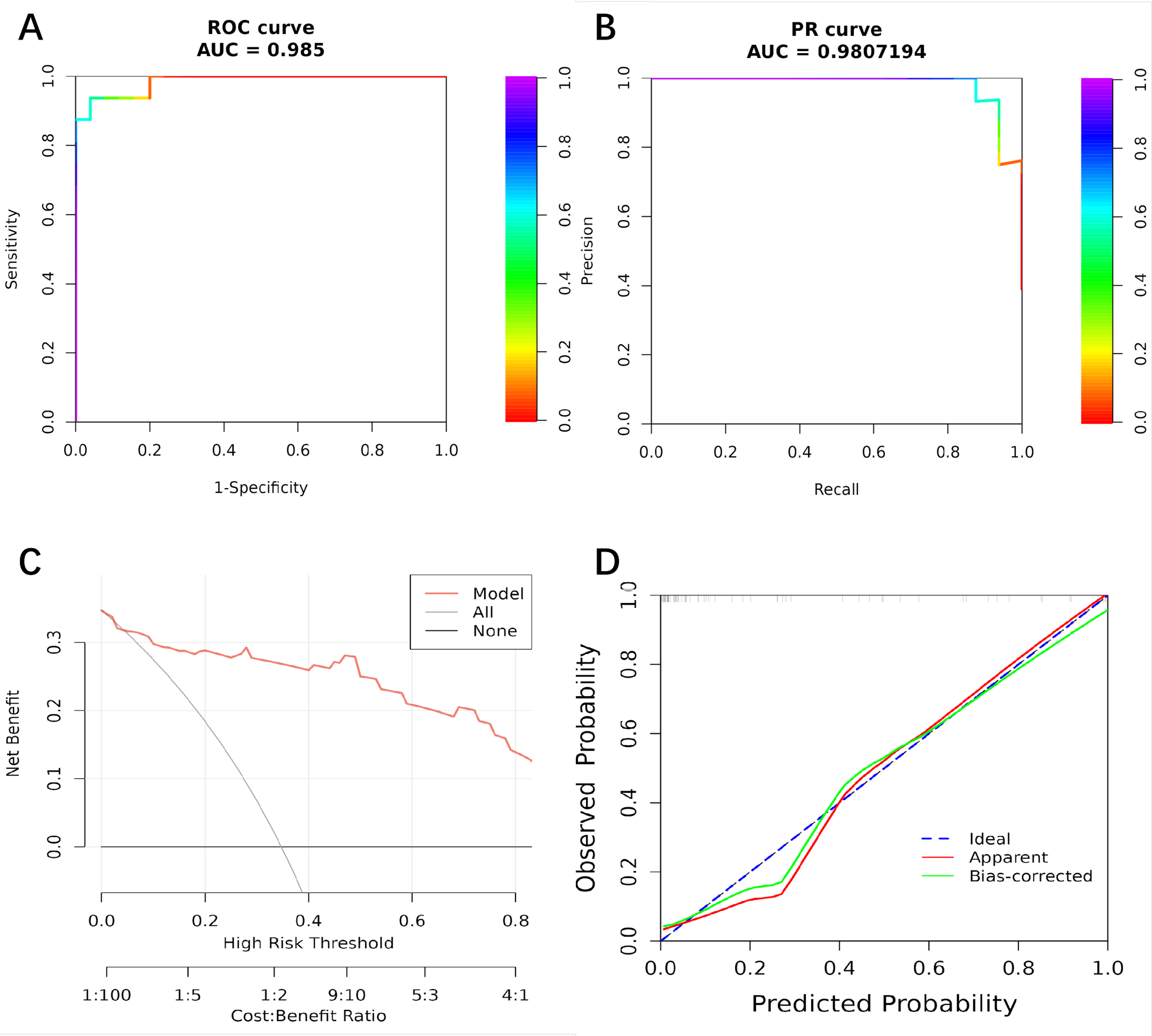


**Figure S4. Performance of the PLTC-nomogram in patients without HCC history (N=72).** (**A**) Receiver operating characteristic (ROC) curve analysis of the nomogram. (**B**) Precision-recall (PR) curve of the nomogram. (**C**) Decision curve analysis (DCA). (**D**) Calibration curves. PLTC, post-liver transplantation complication.


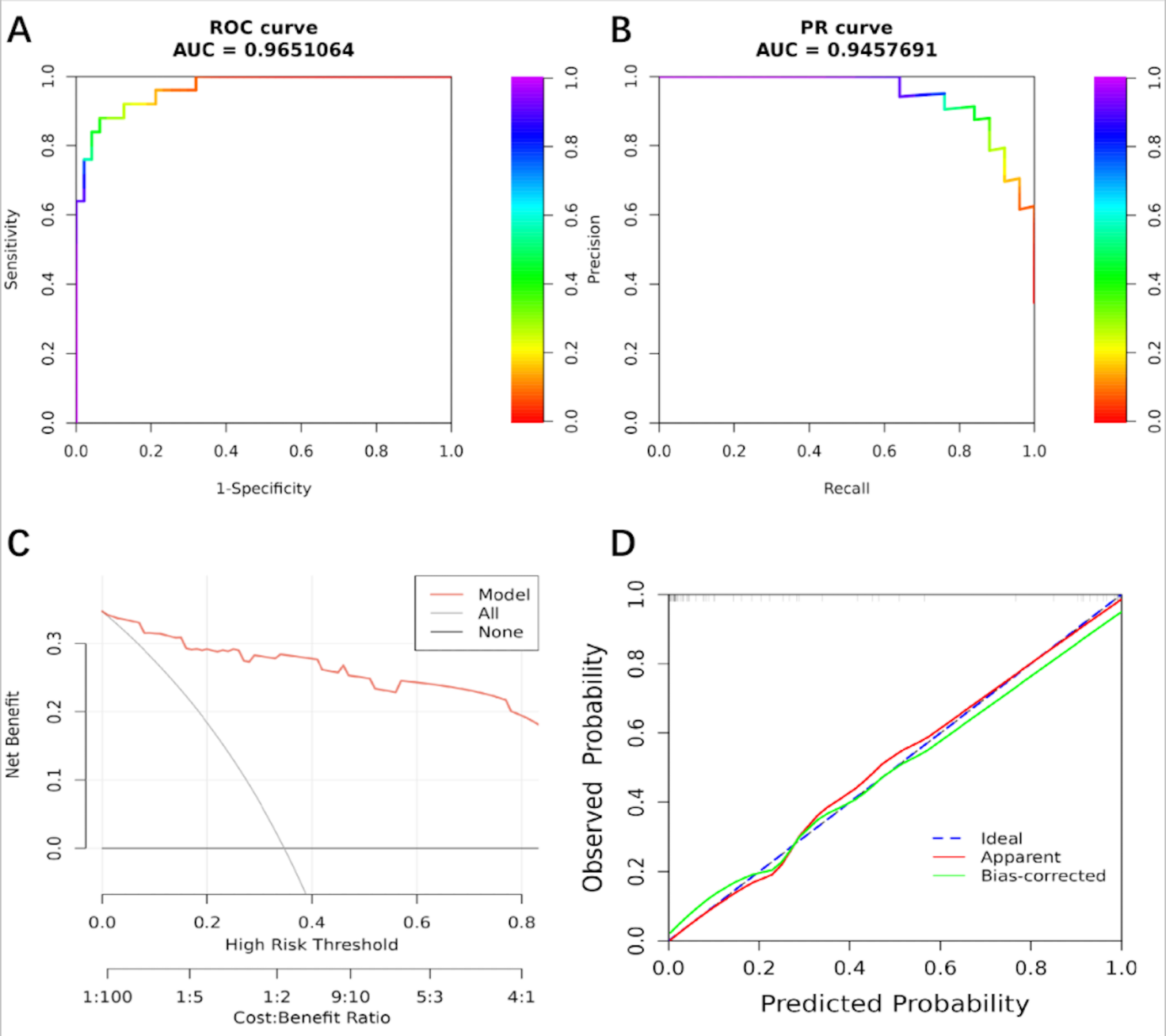

Supplement: Supplementary materials.docx [file IANN_A_2564928_SM1288.docx]
